# A New Transformation Technique for Reducing Information Entropy: A Case Study on Greyscale Raster Images

**DOI:** 10.3390/e25121591

**Published:** 2023-11-27

**Authors:** Borut Žalik, Damjan Strnad, David Podgorelec, Ivana Kolingerová, Luka Lukač, Niko Lukač, Simon Kolmanič, Krista Rizman Žalik, Štefan Kohek

**Affiliations:** 1 Faculty of Electrical Engineering and Computer Science, University of Maribor, Koroška Cesta 46, SI-2000 Maribor, Slovenia; damjan.strnad@um.si (D.S.); david.podgorelec@um.si (D.P.); luka.lukac@um.si (L.L.); niko.lukac@um.si (N.L.); simon.kolmanic@um.si (S.K.); krista.zalik@um.si (K.R.Ž.); stefan.kohek@um.si (Š.K.); 2 Department of Computer Science and Engineering, University of West Bohemia, Technická 8, 306 14 Plzeň, Czech Republic; kolinger@kiv.zcu.cz

**Keywords:** computer science, algorithm, string transformation, information entropy, Hilbert space filling curve

## Abstract

This paper proposes a new string transformation technique called *Move with Interleaving* (MwI). Four possible ways of rearranging 2D raster images into 1D sequences of values are applied, including scan-line, left-right, strip-based, and Hilbert arrangements. Experiments on 32 benchmark greyscale raster images of various resolutions demonstrated that the proposed transformation reduces information entropy to a similar extent as the combination of the Burrows–Wheeler transform followed by the Move-To-Front or the Inversion Frequencies. The proposed transformation MwI yields the best result among all the considered transformations when the Hilbert arrangement is applied.

## 1. Introduction

Information entropy is a measure for an uncertainty in data [1]. Data with lower entropy have reduced diversity and, consequently, are more predictable. The concept was introduced by Shannon [2]. It finds applications in various disciplines including computer science [3], mathematics [4], chemistry [5], mechanics [6], and statistics [7]. In computer science, we are often interested in determining the minimum number of bits required to encode a message *X*, where $X=\langle x_{i}\rangle$ is a sequence of symbols from the alphabet $\sum_{X}=\left\{x_{i}\right\}$. Each symbol $x_{i}\in \sum_{X}$ is assigned a probability $p_{i}$, which is calculated as the ration of the number of occurrences of $x_{i}$ in *X* to the number of all symbols in *X*. Shannon’s information entropy is calculated with Equation (1), and provides a lower bound on the average number of bits required to represent symbols $x_{i}\sum_{X}$.

(1) $$H\left(X\right)=-\sum_{i=0}^{|\sum_{X}|-1}p_{i}\log_{2}\left(p_{i}\right).$$

The entropy is strongly related to the efficiency of various compression algorithms; lower entropy leads to better compression [8,9]. However, there are known techniques that can influence the information entropy of *X* [10], including predictions and transformations. This paper initially considers three such transformation techniques: Move-To-Front, Inversion Frequencies, and the Burrows–Wheeler Transform. A new transformation technique is proposed later.

This paper is divided into five Sections. Section 2 provides a brief explanation of Move-To-Front, Inversion Frequencies, and the Burrows–Wheeler Transform. Section 3 introduces the new transformation method named Move-With-Interleaving (MwI), and discusses various possibilities for arranging the data from the raster images into sequences *X*, which are then transformed. The results of applying the considered transformations on 32 benchmark greyscale raster images are presented in Section 4. Section 5 concludes the paper.

## 2. Background

String transformation techniques, including Move-To-Front [11], Inversion Frequencies [12], Sorted Inversion Frequencies [13], Frequency Count [14], Weighted Frequency Count [15], Distance Coding [16], Time Stamp [17], Burrows–Wheeler Transform [18], have attracted a lot of research attention in the past. In this paper, however, we will limit our focus to the most known techniques, namely Move-To-Front, Inversion Frequencies, Burrows–Wheeler Transform, and their combinations [19].

### 2.1. Move-To-Front Transform

Move-To-Front (MTF) transformation was introduced independently by Ryabko [11], and, shortly thereafter, by Bentley et al. [14]. It is one of the self-organising data structures [20]. MTF transforms $X=\langle x_{i}\rangle$, $x_{i}\in \sum_{X}$, into $Y=\langle y_{i}\rangle$, $y_{i}\in \sum_{Y}=\left\{0,1,2,\cdots ,\right|\sum_{X}\left|-1\right\}$. Not all elements from $\sum_{Y}$ need to exist in *Y*. MTF changes the domain from $\sum_{X}$ to the set of natural numbers including 0. The lengths of the sequences *X* and *Y* remain the same, i.e., |X|=|Y|. MTF utilises a list *L* with random access, and operates through the following steps:

- Initialisation: fill *L* with all $x_{i}\in \sum_{X}$,
- For each $x_{i}\in X$:
  – Find the index *l* in *L* where $x_{i}$ is located;
  – Send *l* to *Y*;
  – Increment the positions of all $x_{k}$, where 0≤k<l;
  – Move $x_{i}$ to the front of *L*.

Let us consider an example in Table 1, where X=〈barbara|barbara〉, $\sum_{X}=\left\{a,\;b,\;r,\;|\right\}$ and H(X)=1.806. MTF transforms *X* into Y=〈1,1,2,2,2,2,1,3,3,2,3,2,2,2,1〉, with H(Y)=1.457. In this example, *Y* contains fewer symbols than $|\sum_{X}|$, although this is not always the case.

MTF reduces the information entropy in data by revealing local correlations. In fact, the sequences of the same symbols are transformed into 0, pairs of symbols are transformed into 1, triplets are transformed into 2, and so on. In some cases, repeated MTF transformations further reduce *H* [21].

The Inverse Move-To-Front (IMTF) Transform is straightforward. The input consists of the sequence of indices $Y=\langle y_{i}\rangle$ and the alphabet $\sum_{X}$. List *L* should be initialised in the same manner as in the MTF case (see Table 1). After that, indices $l=y_{i}$ are taken one by one from *Y*. The symbol $x_{i}$ at index *l* in *L* is read and sent to *X*. *L* is then rearranged in the same way as during the transformation.

### 2.2. Inversion Frequencies

Transformation Inversion Frequencies (IF) was proposed by Arnavut and Magliveras [12,22]. IF accepts $X=\langle x_{i}\rangle$ as an input, where $x_{i}$ is from the alphabet $\sum_{X}$, and transforms it into $Y=\langle y_{i}\rangle$, $y_{i}\in \sum_{Y}$, where $\sum_{Y}=\left\{0,1,2,\cdots ,|X|-1\right\}$. Similarly to MTF, IF transforms the input symbols into the domain of natural numbers, but this time, the limit is |X| instead of $|\sum_{X}|$ as in the case of MTF. Of course, not all elements from $\sum_{Y}$ need to be present in *Y*.

For each $x_{i}\in \sum_{X}$, IF stores the position (i.e., an index) of its first appearance in *X*, and calculates an offset for all subsequent occurrences of $x_{i}$. However, all symbols $x_{j}\in \sum_{X}$, 0≤j<i, that have been used up to this point, are skipped over. The partial results for each $x_{i}$ are stored in auxiliary sequences $A_{x_{i}}$, which are merged in *Y* at the end.

Let us transform X=〈barbara|barbara〉 with IF, where $\sum_{X}=\left\{a,\;b,\;r,\;|\right\}$. The partial transformations are:

- $A_{a}=\langle 1,2,1,2,2,1\rangle$;
- $A_{b}=\langle 0,1,2,1\rangle$;
- $A_{r}=\langle 0,0,1,0\rangle$;
- $A_{|}=\langle 0\rangle$.

The first ‘a’ is located at position 1 in *X* and, therefore, the first entry into $A_{a}$ is 1. To reach the next ‘a’, two symbols (‘r’ and ‘b’) have to be skipped, and therefore, the next entry into $A_{a}$ is 2. The remaining entries in $A_{a}$ are obtained using the same principle. First, ‘b’ is located at index 0. Two symbols (‘a’ and ‘r’) exist before the next ‘b’. However, ‘a’ was already used, giving the offset 1. The first appearance of ‘r’ in *X* is at the position 2. However, as ‘b’ and ‘a’ were already used they should be skipped, and therefore, the first entry in $A_{r}$ is 0. All the auxiliary arrays are then merged into Y=〈1,2,1,2,2,1,0,1,2,1,0,0,1,0,0〉 with H(Y)=1.566. Expectantly, the values in the auxiliary sequences become smaller gradually, with all entries being zero for the last symbol.

Inverse Inversion Frequency (IIF) transformation requires information about the lengths of auxiliary sequences, i.e., the frequencies of the symbols in *X*, in addition to *Y* and $\sum_{X}$. In our example, F=〈6,4,4,1〉. However, *F* could be avoided by the introduction of a guard, which should not be an element in $\sum_{X}$. The guard then separates the elements from auxiliary sequences. As we know that the last auxiliary array only contains zeros, it can be avoided. If the guard is −1, then Y=〈1,2,1,2,2,1,−1,0,1,2,1,−1,0,0,1,0,−1〉. When the occurrence of the last symbol exceeds $|\sum_{X}|$, |Y|<|X| could be advantageous, for example, for compression.

### 2.3. Burrows–Wheeler Transform

One of the ideas on how to transform *X* could be the generation of all possible permutations, and then selecting the one with the highest local correlations. The consecutive number of this permutation should be stored to reproduce the *X*. Unfortunately, the number of permutations grows exponentially by |X|, and this approach is, therefore, not applicable in practice. However, one of the permutations is obtained by sorting. The sorted sequence offers many good properties; among others, the local correlations are also emphasised. Unfortunately, an inverse transformation, which would convert the sorted sequence into its unsorted source, is not known. Burrows–Wheeler Transform (BWT), one of the most surprising algorithms in Computer Science [23], constructs the permutation of *X*, where the same symbols tend to be close together. In addition, only O(1) additional information is needed to restore *X*. Transformation, as suggested by Burrows and Wheeler [18], consists of four steps:

1. Generating |X| permutations of *X* through rotational shift-right operations;
2. Sorting the obtained permutations lexicographically;
3. Reading the BWT(*X*) from the last column of the sorted permutations;
4. Determining the position of *X* in the sorted array of permutations. This position is essential for reconstruction, and is considered a BWT index, $i_{BWT}$;

The construction of BWT for X=〈barbara|barbara〉 is shown in Table 2. The majority of the same symbols are placed together in the obtained result Y=〈rbbbbrrr|aaaaaa〉. The position of *X*, $i_{BWT}=9$, should be stored for reconstruction.

$i_{BWT}=9$ is needed to reconstruct *X* from *Y*. The first column *C* of the sorted array of permutations is obtained from *Y* straightforwardly by sorting (see Table 3). The first symbol is easily obtained from *C*, as it is pointed by $i_{BWT}=9$, $C_{9}=‘b’$ and X=〈b〉. The symbol ‘b’ is the fourth ‘b’ in *C*, and, therefore, it can be found in *Y* at the position 4. $C_{4}=‘a’$ is inserted into X=〈ba〉. The found symbol was the fifth ‘a’ in *C*, so the fifth ‘a’ is searched for in *Y*. It is found at position 13, where the corresponding $C_{13}$=‘r’ is added into X=〈bar〉. The process continues until index $i_{BWT}$ is reached again.

## 3. Materials and Methods

Continuous-tone greyscale raster images (i.e., photographs) are used in our study, and therefore, the new transformation technique, introduced in Section 3.1, is designed accordingly. There are various transformations commonly applied to images among with Discrete Cosine Transform (DCT) and Discrete Wavelet Transform (DWT) among the most widely used. These transformations are most frequently used for the spectral analysis of the image for the quantification of higher frequencies for lossy compression. The rare exception is JPEG2000 with LGT 5/3 wavelet which enables lossless compression. However, previous studies demonstrate higher compression ratios for more advanced prediction-based encoders for lossless compression, such as JPEG-LS [24,25], FLIF, and JPEG XL lossless [26]. Prediction methods are more commonly used for reducing information entropy for lossless image compression [27]. However, these methods are domain-dependent, whereas the proposed transformation methods are general.

Images are 2D structures that should be rearranged into 1D sequences to apply the aforementioned transformation techniques. However, this rearrangement can be performed in different ways, as discussed in Section 3.2.

### 3.1. Move with Interleaving

Let *I* be a greyscale raster image consisting of pixels $p_{x,y}$, 0≤x<N, 0≤y<M, where N×M is the image resolution, and $p_{x,y}\in \left[0,255\right]$. The pixels are arranged in the sequence $X=\langle x_{i}\rangle$, $x_{i}\in \left[0,255\right]$, |X|=N×M, on which transformation is applied in order to reduce the information entropy.

Neighbouring pixels $p_{x,y}\in I$, and therefore also consecutive symbols $x_{i}\in X$, often reveal local correlations. However, in the majority of cases in photographs, these correlations do not manifest as the sequences of the same values or repeated patterns; instead, these values tend to be just *similar enough* within a certain tolerance δ. It can be assumed that the suitable value for δ depends on the specific image, and therefore, it is experimentally determined in Section 4. The values of $x_{i}$ change importantly only when the scene in *I* changes drastically (for example, such as during the transition from a branch of a tree the image background [28]). MTF would, in such a case (see Section 2.1), issue a long sequence of large indices to bring enough similar values near to the beginning of the list. Unfortunately, this means that the values in *Y* would be considerably dispersed, which is undesirable from the information entropy perspective.

The proposed transformation is based on the idea of MTF and, therefore, utilises list *L* with random access. Let $x_{i}\in X$ represent the value to be transformed. The position *l* of $x_{i}$ in *L* should be found and *l* is sent to *Y*. The updating process of the *L* operates in two modes:

Mode 1: MTF is applied when l≤δ.
Mode 2: The temporal array *T* is filled with 2·δ interleaved values, starting with $x_{i}$ when l>δ. The values from *T* are then inserted at the front of *L*, shifting all the remaining values in *L* accordingly. This is why we named this transformation *Move with Interleaving* (MwI).

Algorithm 1 presents a pseudocode for the MwI transform, which is demonstrated by an example, where the alphabet $\sum_{X}\in \left[0,15\right]$. Given X=〈7,9,11,10,2,⋯〉 as the first five elements of *X*, and let δ=3, the following steps are performed:

1. Function InitialiseL in Line 4 of Algorithm 1 obtains the first element from *X* ($x_{0}=7$) and δ=3 as input, and populates the list *L*. The first element in *L* becomes $x_{0}$, while 2·δ elements from interval [7−3,7+3] are interleaved around this value. The remaining elements of *L* are then filled from the smallest up to the largest possible value from the interval [0,15], according to the alphabetical order in $\sum_{X}$. $x_{0}$ also becomes the first element in *Y* (see Line 5) to enable the same initialisation of *L* during the decoding phase. The situation after the initialisation is therefore:
  i=0
  X=〈7,9,11,10,2,⋯〉
  L=〈7,8,6,9,5,10,4,0,1,2,3,11,12,13,14,15〉.
  Y=〈7〉
2. The remaining elements from *X* are then transformed within the For-loop starting at Line 6 and ending in Line 15. Let us follow the algorithm for some elements from *X*.
  i=1: Function in Line 7 finds the position l=3 for $x_{1}=9$. *l* is inserted into *Y* in Line 8. The MTF transform in Line 10 is applied as l≤δ to obtain the following situation:
    i=1
    X=〈7,9,11,10,2,⋯〉
    L=〈9,7,8,6,5,10,4,0,1,2,3,11,12,13,14,15〉.
    Y=〈7,3〉
  i=2: For $x_{2}=11$, function GetPosition returns l=11, which is inserted into *Y*. As l>δ, function FillT in Line 12 generates the interleaved values around $x_{2}=11$. It returns T=〈11,12,10,13,9,14,8〉. Function ModifyL in Line 13 moves *T* in front of *L*, while other values are placed after it. We obtained:
    i=2
    X=〈7,9,11,10,2,⋯〉
    T=〈11,12,10,13,9,14,8〉
    L=〈11,12,10,13,9,14,8,7,6,5,4,0,1,2,3,15〉.
    Y=〈7,3,11〉
  i=3: GetPosition returns l=2 for $x_{3}=10$ and inserts *l* into *Y*. Function MTF is applied because l<δ. The obtained situation is:
    i=3
    X=〈7,9,11,10,2,⋯〉
    L=〈10,11,12,13,9,14,8,7,6,5,4,0,1,2,3,15〉.
    Y=〈7,3,11,2〉
  i=4: $x_{4}=2$, l=13, l>δ. Sequence T=〈2,3,1,4,0,5〉 contains only 6 elements this time, as the values being outside [0,15] are not inserted. The obtained situation is therefore:
    i=4
    X=〈7,9,11,10,2,⋯〉
    T=〈2,3,1,4,0,5〉
    L=〈2,3,1,4,0,5,10,11,12,13,9,14,8,7,6,15〉.
    Y=〈7,3,11,2,13〉

**Algorithm 1** Transformation MwI1:**function** MwI(*X*, δ)▹ Returns transformed sequence *Y*2:
▹ *X*: input sequence; δ: tolerance3: i=04:  L=▹ InitialiseL($x_{i}$, δ)Initialisation of *L* is done according to $x_{i=0}$5:  $Y_{0}=x_{i}$▹ The first entry in *Y* is $x_{i=0}$6: **for** i←1
**to**
|X|
**do**▹ For all other $x_{i}$7:  l= GetPosition(*L*, $x_{i}$)▹ Find the position of $x_{i}$ in *L*8:  Y= AddToY(*Y*, *l*)▹ Store the position in *Y*9:  **if** l≤δ
**then**▹ If position is smaller that δ10:   L= MTF(*l*, *L*)▹ Then rearrange *L* according to MTF11:  **else**▹ otherwise12:   T= FillT($x_{i}$, δ)▹ Fill temporal sequence *T*13:   L= ModifyL(*L*, *T*)▹ Place symbols in *T* in front of *L*14:  **end if**15: **end for**16: **return**
*Y*▹ Returns transformed sequence17:
**end function**


The inverse MwI transformation (IMwI) is shown in Algorithm 2. As can be seen, it completely mimics the transformation procedure. The first element in *Y* represent the absolute value of $x_{0}$, and it is obtained in Line 3. $x_{0}$ is utilised to populate the list *L* in Line 4, and depended on the output sequence *X* in Line 5. All other elements in *Y* are processed with the *for-loop* starting in Line 6. The specific position *l* is obtained from *Y* (Line 7), the value *v* is retrieved from *L* at the position *l* (Line 8), and stored in *X* in Line 9. After that, the algorithm evaluates *l* with regard to δ and applies either MTF (Line 11) or resets the content of *L* in Lines 13 and 14. When all indices from *X* have been processed, the reconstructed values are returned in Line 17.

**Algorithm 2** Inverse MwI transformation1:**function** IMwI(*Y*, δ)▹ Returns restored sequence *X*2:▹ *Y*: input sequence of indices; δ: tolerance3: $x_{0}=Y_{0}$▹ The first entry in *X* is $Y_{0}$4: L= InitialiseL($x_{0}$, δ)▹ Initialisation of *L* is done according to first element5: AddToX(*X*, $x_{0}$)▹$x_{0}$ is sent to the reconstructed sequence *X*6: **for** i←1
**to**
|Y|−1
**do**▹ For all other $y_{i}$7:  $l=Y_{i}$▹ Get the position from *Y*8:  $v=L_{l}$▹ Get the value from *L*9:  X= AddToX(*X*, *v*)▹ Store the value in *X*10:  **if** l≤δ
**then**▹ If position is smaller that δ11:   L= MTF(*l*, *L*)▹ Then rearrange *L* according to MTF12:  **else**▹ Otherwise13:   T= FillT(*v*, δ)▹ Fill temporal sequence *T*14:   L= ModifyL(*L*, *T*)▹ Place symbols in *T* in front of *L*15:  **end if**16: **end for**17: **return**
*X*▹ Returns restored sequence18:
**end function**


#### Time Complexity Estimation

The worst-case time complexity analysis for the considered transformation techniques is performed in this subsection.

MTF: In the worst-case scenario, the last element of *L* should always be moved to the front. There are $|\sum_{X}|$ elements in *L* and consequently, $T_{MTF}\left(X\right)=|\sum_{X}\left|\cdot |X\right|$. Since $|\sum_{X}\left|<<|X\right|$, $T_{MTF}\left(X\right)=O\left(|X|\right)$.
IF: For each $x_{i}\in \sum_{X}$, all elements in *X* are always visited, resulting in $T_{IF}\left(X\right)=|\sum_{X}\left|\cdot |X\right|$. Again, since $|\sum_{X}\left|<<|X\right|$, $T_{IF}\left(X\right)=O\left(|X|\right)$.
BWT: The algorithm presented in Section 2.3 has, unfortunately, A time complexity of $O(|X{|}^{2}\log\left|X\right|)$, which limits its practical use for longer sequences. Later, it was shown that BWT can be constructed from the suffix array in linear time [23], and since there are known algorithms for constructing the suffix array in linear time [29,30], BWT itself can be obtained in $T_{BWT}\left(X\right)=O\left(|X|\right)$ time.
BWT+MTF: Based on the above analysis, the combination BWT, followed by MTF, works in $T_{BWT+MTF}\left(X\right)=T_{BWT}\left(X\right)+T_{MTF}\left(X\right)=O\left(|X|\right)$.
BWT+IF: Similarly, as above, the combination BWT, followed by IF, operates in time complexity $T_{BWT+IF}\left(X\right)=T_{BWT}\left(X\right)+T_{IF}\left(X\right)=O\left(|X|\right)$.
MwI: MwI operates in two modes. In mode 1, then $T_{MwI}\left(X\right)=T_{MTF}\left(X\right)=O\left(|X|\right)$. In mode 2, the algorithm performs two tasks. Firstly, it fills the auxiliary sequence *T* with Δ=1+2·δ elements. After that, it applies MTF Δ times resulting in a total of $T_{MwI}\left(X\right)=\Delta \cdot |\sum_{X}\left|\cdot |X\right|$ operations. Since $\Delta \leq |\sum_{X}\left|<<|X\right|$, $T_{MwI}\left(X\right)=O\left(|X|\right)$.

### 3.2. Rearranging Raster Data in the Sequence

**Figure 1:**
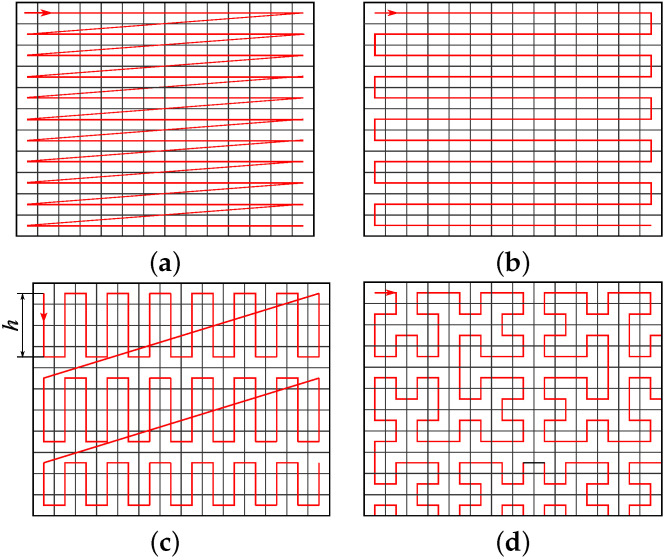
Different arrangements of pixels into a sequence: (**a**) Scan-line; (**b**) Left-right; (**c**) Strip; and (**d**) Hilbert.

Images are typically rearranged into sequences using a Scan-line order, which is a heritage of television (see Figure 1a). Three other possibilities shall be used for our experiments:

- Left-right scan (Figure 1b);
- Strip scan (Figure 1c);
- Hilbert scan (Figure 1d).

The Strip arrangement requires a user-defined parameter *h* for the width of the strip. Its value is evaluated in Section 4. A well-established approach for transforming multidimensional data into a one-dimensional form is through the use of space-filling curves. The Hilbert curve [31] has been frequently applied to images [10,32,33]. An implementation based on the state diagram [34] has been used for mapping between 2D images and 1D sequences, and vice versa. The complete Hilbert curve can only be constructed on images with resolutions equal to powers of 2 in both directions. However, images of different resolutions are quite common. Therefore, the Hilbert curve is cut off accordingly, as shown in Figure d.

## 4. Experiments

**Figure 2:**
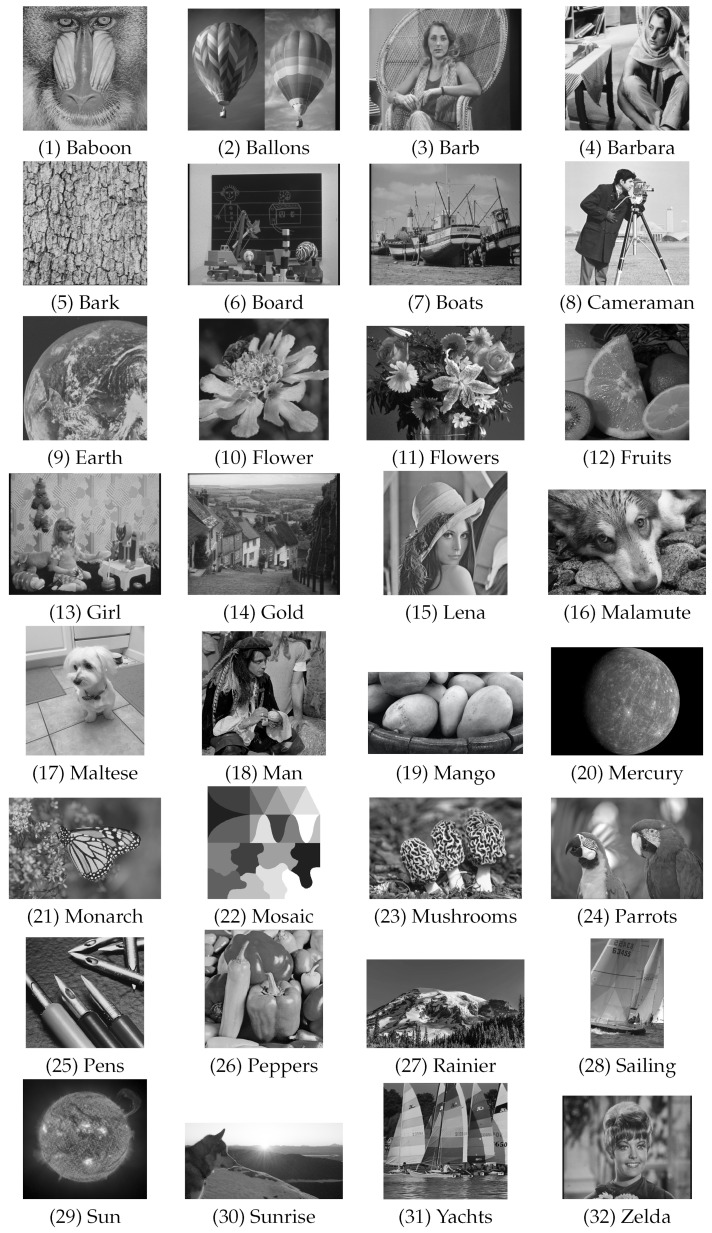
Testing raster images.

Figure 2 shows 32 benchmark 8-bit greyscale images used in the performed experiments. Table 4 gives the resolutions of these images in the second column, and their information entropies in the third one.

The information about the proposed transformation MwI is given in the fourth and fifth columns: firstly, the best values of δ, and secondly, the achieved information entropies. On average, the best value of δ is 12. However, δ=11 was used for further experiments, since 17 out of the 32 images achieved the best reduction in entropy with δ<12. The decrease in information entropy is shown in columns 6, 7, and 8 of Table 4 for MTF, IF, and MwI, respectively. MwI considerably outperformed MTF and IF.

BWT was used before MTF, IF, and MwI in the last three columns of Table 4. BWT had a considerably positive effect only on MTF and IF, but not on MwI. MwI, with its transformation mechanism, is capable of entirely replacing BWT. The last row of Table 4 shows the rank achieved by all the considered transformations. The ranking was as follows: BWT in front of IF was in the first place, MwI was in the second, BWT followed by MTF was in the third, while MwI after BWT, IF, and MWI were in the fourth, fifth, and the sixth places, respectively.

**Table 4:** Information about the images’ resolutions and their entropies *H*, and the entropies obtained by different transformations—all for the Scan-line order.

*I*	*N* × *M*	*H*	$\delta_{\mathrm{opt}}$	**MwI($\delta_{\mathrm{opt}}$)**	MTF	IF	MwI ^1^	BWT MTF	BWT IF	BWT MwI ^1^
(1)	512×512	7.357	25	6.471	7.308	7.415	6.567	6.656	6.680	6.661
(2)	720×576	7.346	5	4.074	5.649	5.497	4.172	3.989	3.920	4.008
(3)	720×576	7.484	13	6.033	6.936	6.868	6.037	6.285	6.112	6.248
(4)	512×512	7.343	16	5.351	6.769	6.888	6.370	6.156	6.020	6.518
(5)	512×512	7.325	40	6.757	7.517	7.849	7.016	6.792	6.873	6.786
(6)	720×576	6.828	9	4.526	5.454	5.364	4.535	4.617	4.459	4.625
(7)	720×576	7.088	11	5.043	6.008	5.909	5.043	5.266	5.095	5.271
(8)	256×256	6.904	11	5.551	6.349	6.323	5.551	5.834	5.667	5.799
(9)	512×512	7.155	17	5.544	6.740	6.702	5.598	5.569	5.511	5.555
(10)	512×480	7.410	8	4.764	6.375	6.288	4.803	4.553	4.463	4.515
(11)	500×362	7.305	17	5.608	6.832	6.668	5.641	5.823	5.629	5.808
(12)	512×480	7.366	10	4.840	6.294	6.218	4.844	4.805	4.657	4.740
(13)	720×576	7.288	12	5.111	6.489	6.455	5.114	5.166	5.067	5.178
(14)	720×576	7.530	14	5.262	6.485	6.308	5.268	5.516	5.382	5.547
(15)	512×512	7.348	10	5.184	6.629	6.720	5.189	5.240	5.118	5.233
(16)	1616×1080	7.792	14	5.427	6.994	6.865	5.439	5.326	5.153	5.321
(17)	2238×2446	6.964	6	3.912	5.056	4.932	4.036	3.927	3.961	4.051
(18)	1024×1024	7.524	15	5.480	6.900	6.834	5.511	5.662	5.523	5.682
(19)	1360×732	7.729	6	4.138	5.739	5.673	4.204	4.060	3.930	4.083
(20)	732×529	4.711	16	3.707	4.352	4.313	3.728	3.850	3.687	3.854
(21)	768×512	7.18	8	4.770	6.082	5.935	4.787	4.770	4.632	4.768
(22)	512×512	2.983	0	0.125	0.125	0.173	0.133	0.133	0.126	0.143
(23)	481×321	7.585	12	6.042	7.230	7.095	6.042	5.961	5.788	5.875
(24)	768×512	7.256	7	4.460	5.842	5.652	4.482	4.590	4.455	4.638
(25)	512×480	7.482	13	5.203	6.977	6.845	5.216	4.908	4.819	4.846
(26)	512×512	7.594	14	5.258	6.840	6.967	5.288	5.459	5.373	5.451
(27)	1920×1080	7.088	21	4.632	5.127	5.022	4.658	4.732	4.510	4.711
(28)	512×768	7.131	9	4.774	5.865	5.677	4.775	4.966	4.845	5.039
(29)	2100×2034	6.950	5	3.599	4.533	4.261	3.641	3.551	3.360	3.713
(30)	6000×2908	7.328	8	4.290	4.741	4.523	4.310	4.411	4.269	4.493
(31)	512×480	7.560	8	5.093	6.421	6.473	5.105	5.098	4.976	5.025
(32)	720×576	7.334	11	4.830	6.536	6.436	4.830	4.941	4.889	4.975
Average		7.102	12.2	4.870	6.039	5.973	4.935	4.949	4.842	4.974
Rank					6	5	2	3	1	4

^1^ δ=11 was used.

Table 5 presents the average entropy of all 32 benchmark images when different pixel arrangements were used to obtain sequence *X*. The results are quite intriguing, and deserve further analysis. For example, MTF significantly benefited from the Strip order, but performed poorly on the Scan-line and Left-right orders. The same pattern also applies to IF. On the other hand, the effect of the arrangement type was reduced when BWT was used in front of MTF or IF. Even more, BWT followed by MTF or IF was the best when the Scan-line order was applied. The pipeline BWT followed by MwI yielded worse results compared to using MwI alone. Therefore, it can be concluded that MwI efficiently replaced BWT. It can be observed that MwI was also not very sensitive to the data arrangements. However, the Hilbert arrangement was the most suitable, as, in this case, MwI achieved the best result between all the tested transformation and data arrangements.

**Table 5:** Average entropies achieved for different arrangements of the pixels in sequences.

**Order**	**MTF**	**IF**	**MwI**	**BWT MTF**	**BWT IF**	**BWT MwI**
Scan-line	6.039	5.973	4.935	4.949	4.842	4.974
Left-right	6.004	5.905	4.925	4.959	4.843	4.977
Strips	5.111 ^1^	5.036 ^2^	4.962 ^3^	5.118 ^4^	5.000 ^4^	5.149 ^1^
Hilbert	5.349	5.905	4.754	5.012	4.889	5.050

^1^ width of the strip h=4. ^2^ width of the strip h=12. ^3^ width of the strip h=8. ^4^ width of the strip h=16.

Besides the formal analysis provided in Section 3.1, it is even more important to consider how efficient the algorithm is in practice. Table 6 shows the CPU time spent on three techniques, all achieving a similar reduction in information entropy for seven images ranging from the smallest to the largest. The Scan-line order was used, and MwI was consistently the fastest in all cases.

**Table 6:** The CPU time spent in seconds for three transformation techniques, all achieving similar reductions in information entropy.

**Image**	**No. of Pixels**	**BWT and MTF**	**BWT and IF**	**MwI**
(8)	65,536	0.042	0.068	0.037
(1)	262,144	0.260	0.308	0.162
(6)	414,720	0.432	0.465	0.217
(18)	1,048,576	1.365	1.589	0.616
(16)	1,745,280	2.554	2.781	0.992
(27)	2,073,600	8.910	9.451	2.901
(30)	17,448,000	31.966	32.669	9.331

Personal computer with AMD Ryzen 5 5500 processor clocked at 3.60 GHz and equipped with 32 GB of RAM, running the Windows 11 operating system, was used in the experiments. The algorithms were programmed in C++ using MS Visual Studio, version 17.4.2.

## 5. Discussion

This paper introduces a transformation technique named *Move with Interleaving* (MwI). It operates in two modes. The first mode is the classical Move-To-Front, where the considered symbol $x_{i}$ from the alphabet $\sum_{X}$ is moved to the front of the list *L*. In the second mode, MwI moves 2·δ symbols interleaved around $x_{i}$ in front of *L*. As a result of MwI, less oscillating transformed values are obtained, which exhibit lower information entropy. The approach proves to be especially beneficial in the sequences of symbols where local correlations are manifested as similar symbols within a certain tolerance, rather than as completely identical symbols, or symbols that reveal repeating patterns. Continuous-tone raster images are typical examples of such data, and were used in this paper to illustrate the concept.

Pixels, which define a raster image, can be arranged into a sequence in various ways, with Scan-order being used the most frequently. Three other possibilities have been tried in this study: Left–right, Strip, and the Hilbert arrangement. The proposed MwI was compared against Move-To-Front (MTF) and Inversion Frequencies (IF) transformations, both individually, and after applying the Burrows–Wheeler transform (BWT).

A total of 32 benchmark 8-bit greyscale raster images with different resolutions and contexts were used in the experiments. The effect of the aforementioned transformations on the information entropy can be summarised as follows:

- When BWT is not used before MwI, it is considerably more efficient than MTF and IF.
- MwI is as efficient as BWT, followed by MTF or IF.
- BWT followed by MwI yields worse results in comparison to the results obtained by MwI alone.
- MwI is less sensitive to the arrangements on the input data compared to MTF and IF.
- MwI is the most efficient transformation technique when the Hilbert data arrangement is used.
- BWT, for its operations, requires the knowledge of the whole sequence in advance, while MwI operates incrementally and can, therefore, also be used in streaming applications.
- Implementing MwI is easier compared to BWT, as it does not require the implementation of a prefix array for computational efficiency.

At this point, it is worth mentioning that string transformation techniques are less efficient than the modern prediction methods in the domain of 2D continuous-tone raster images [27,35]. In future work, it would be interesting to investigate the combination of prediction-based methods and the proposed MwI transformation. A comprehensive comparison with other string transformation techniques and data domains, such as audio, should be conducted. And finally, open challenges remain: how to set δ for each individual data sequence, or even better, how to dynamically modify it during the processing of the considered data sequences.

## Figures and Tables

**Table 1:** MTF Transform: an example.

*X*		/ ^1^	b	a	r	b	a	r	a	|	b	a	r	b	a	r	a
*L*	0	a	b	a	r	b	a	r	a	|	b	a	r	b	a	r	a
	1	b	a	b	a	r	b	a	r	a	|	b	a	r	b	a	r
	2	r	r	r	b	a	r	b	b	r	a	|	b	a	r	b	b
	3	|	|	|	|	|	|	|	|	b	r	r	|	|	|	|	|
*Y*			1	1	2	2	2	2	1	3	3	2	3	2	2	2	1

^1^ Initialisation.

**Table 2:** BWT: an example.

i	Step 1	Step 2	Step 3	Step 4
0	barbara|barbara	abarbara|barbar	r	
1	arbara|barbarab	arabarbara|barb	b	
2	rbara|barbaraba	ara|barbarabarb	b	
3	bara|barbarabar	arbarabarbara|b	b	
4	ara|barbarabarb	arbara|barbarab	b	
5	ra|barbarabarba	a|barbarabarbar	r	
6	a|barbarabarbar	barabarbara|bar	r	
7	|barbarabarbara	bara|barbarabar	r	
8	barbarabarbara|	barbarabarbara|	|	
9	arbarabarbara|b	barbara|barbara	a	←
10	rbarabarbara|ba	rabarbara|barba	a	
11	barabarbara|bar	ra|barbarabarba	a	
12	arabarbara|barb	rbarabarbara|ba	a	
13	rabarbara|barba	rbara|barbaraba	a	
14	abarbara|barbar	|barbarabarbara	a	

**Table 3:** Reconstructing BWT.

*i*	0	1	2	3	4	5	6	7	8	9	10	11	12	13	14
*Y*	r	b	b	b	b	r	r	r	|	a	a	a	a	a	a
*C*	a	a	a	a	a	a	b	b	b	b	r	r	r	r	|

## Data Availability

The data presented in this study are available on request from the corresponding author.
